# Incidence and Molecular Characterization of Hepatitis E Virus from Swine in Eastern Cape, South Africa

**DOI:** 10.1155/2017/1073253

**Published:** 2017-01-16

**Authors:** Olusesan Adeyemi Adelabu, Benson Chuks Iweriebor, U. U. Nwodo, Larry Chikwelu Obi, Anthony Ifeanyi Okoh

**Affiliations:** ^1^SAMRC Microbial Water Quality Monitoring Centre, University of Fort Hare, Private Bag X1314, Alice, Eastern Cape Province 5700, South Africa; ^2^Applied and Environmental Microbiology Research Group (AEMREG), Department of Biochemistry and Microbiology, University of Fort Hare, Private Bag X1314, Alice, Eastern Cape Province 5700, South Africa; ^3^Academic and Research Division, University of Fort Hare, Private Bag X1314, Alice, Eastern Cape Province, South Africa

## Abstract

Hepatitis E virus-mediated infection is a serious public health concern in economically developing nations of the world. Globally, four major genotypes of HEV have been documented. Hepatitis E has been suggested to be zoonotic owing to the increase of evidence through various studies. Thus far, this paper reports on prevalence of hepatitis E virus among swine herd in selected communal and commercial farms in the Eastern Cape Province of South Africa. A total of 160 faecal samples were collected from swine herds in Amathole and Chris Hani District Municipalities of Eastern Cape Province for the presence of HEV. Of the 160 faecal samples screened, only seven were positive (4.4%) for HEV. The nucleotide sequences analyses revealed the isolates as sharing 82% to 99% identities with other strains (KX896664, KX896665, KX896666, KX896667, KX896668, KX896669, and KX896670) from different regions of the world. We conclude that HEV is present among swine in the Eastern Cape Province, albeit in low incidence, and this does have public health implications. There is a need for maintenance of high hygienic standards in order to prevent human infections through swine faecal materials and appropriate cooking of pork is highly advised.

## 1. Introduction

In many developing countries in Asia, Middle East, and Africa, Hepatitis E has become a significant public health concern [1, 2], as well as sporadic cases of acute hepatitis in developed countries [3]. It is a generic term symbolising infection of the liver which is caused by different viruses such as hepatitis A to hepatitis E [4].

Hepatitis E virus is still considered an emerging pathogen in developing countries, owing to the scarcity of information on its infection especially in animals which have arisen from few studies and neglect of the disease and its public health problem [5]. Although most of available information describe human HEV outbreaks in Kenya, Sudan, Uganda, Democratic Republic of Congo (DRC), and Central African Republic [6, 7], information on HEV infection in animals in developing countries of Africa remains underreported.

The widespread HEV infection has been reported in Nigeria, with prevalence rate of 76.7% of genotype 3, and also in Cameroon, Republic of Congo, and Tunisia [8]. The causative agent of the infection known as hepatitis E virus, is a nonenveloped, single-stranded, and positive sense RNA virus, with nearly 7.2 kb genome which encodes three open reading frames (ORFs), translated into ORFs 1–3, with a short 59 untranslated region (UTR). Open reading frame 1 encodes for nonstructural protein while ORF 2 and ORF 3 encode for viral capsid protein and multifunctional small proteins, respectively [9]. It was classified as the only member of the genus* Hepevirus *[10]. However, a new taxonomic division within the family Hepeviridae, due to contradictions in respect to the designation of species and genotypes, has been proposed by Smith et al. (2014), in which the family Hepeviridae is divided to genera* Orthohepevirus* (all mammalian and avian HEV isolates) and* Piscihepevirus *(cutthroat trout virus) while species within the genus* Orthohepevirus* are assigned* Orthohepevirus* A-D [11, 12].

Several studies have reported HEV to be transmitted via faecal-oral routes, usually via contaminated water in developing countries [13–15], while, in the developed countries, direct contacts with infected animals and consumption of poorly cooked/contaminated pork meat and its products, among others, have been reported as routes of transmission and are probable means of zoonotic HEV transmission [16, 17]. The recent identification of other various genetically diverse* hepatitis E virus* strains from different animal species poses additional potential concerns for HEV zoonotic infection [12, 18]

HEV is a single serotype virus with four distinct genotypes which have the potential of infecting man (genotypes I–4). Genotypes 1 and 2 are primarily classified to infect only human population, while genotypes 3 and 4 are said to be zoonotic in developing and developed nations [19–22].

Though it is a slight to temperate disease in severity with mortality rate of <1–4%, among young adults but greater in infected pregnant women, where high mortality rate is progressive in each succeeding trimester to approximately 40%, about 90% of children aged <10 years infected with HEV living in HEV endemic areas have been reported [23].

Several decades into the documentation of the Hepatitis E virus genome, medical virology research is still advancing in knowledge acquisition sequel to the amplified alertness and apparent prominence of hepatitis E as a significant public health concern [16]. Analyses of HEV from animal reservoirs have also confirmed that the strains circulating among domestic and wild pigs are genetically related to the strains identified in human cases [12, 24]. In this paper, we report on the prevalence of swine HEV isolates in selected swine herds in Eastern Cape, South Africa, using the highly conserved capsid protein.

## 2. Materials and Methods

### 2.1. Description of the Study Areas

Both commercial and communal swine herds from Amathole and Chris Hani Districts Municipalities were selected for this study, and, for the purpose of confidentiality, these herds will be referred to as swine herds A and B.

Swine herd A is located along the geographical coordinates of 31°34′0′′S and 28°46′0′′E. It has about 1000 pigs penned in groups of 8 to 10 according to sizes and age.

Swine herd B is located along the geographical coordinates of 32°30′0′′S and 27°30′0′′E, with capacity of approximately 400 pigs, ranging from nursery to finisher pigs in different pens.

## 3. Sample Collection

One hundred and sixty faecal samples from 3–5-month-old pigs in selected swine herds were collected with sterile swab for this study. Ten (10 grams) of the samples were each separately suspended in 1x Phosphate buffered saline (PBS) followed by vigorous shaking with vortex machine, centrifuged at 5000 rpm for 5 min, and the supernatants were separated and stored at −80°C until further use as source of HEV genomic RNA.

## 4. Viral RNA Extraction

The Viral RNA was purified from 160 *µ*l of the swine faecal samples using Quick-RNA Miniprep kit (Zymo research, USA), the manufacturer's protocol was adhered to. The Viral RNA was eluted from the spin column with 50 *µ*l of the elution buffer and frozen at −80°C until further use.

## 5. Nested Reverse-Transcriptase Polymerase Chain Reaction (RT-PCR)

Two sets of degenerated primers flanking a segment of the ORF 2 gene encoding the structural capsid protein were used: external primers [Forward 5′-AGCTCCTGTACCTGATGTTGACTC-3′], [Reverse 5′-CTACAGAGCGCCAGCCTTGATTGC-3′] with expected RT-PCR product size of 427 bp and internal primers [Forward, 5′-GCTCACGTCATCTGTCGCTGCTGG-3′], [Reverse, 5′-GGGCTGAACCAAAATCCTGACATC-3′] with expected RT-PCR product size of 289 bp.

The RNA was reverse transcribed using 5 *µ*l of total RNA, 1 *µ*l each of both forward and reverse primers (external primers), 12 *µ*l of master-mix, 5.6 *µ*l of RNase-free water, and 0.4 *µ*l of reverse transcriptase (Thermo-Fisher, Scientific), at 50°C for 60 min to generate complementary DNA (cDNA), followed by a conventional PCR using 5 *µ*l of cDNA as template with master-mix: 12 *µ*l, forward and reverse primers: 1 *µ*l each, and RNase-free water: 6 *µ*l to make a final reaction volume of 25 *µ*l, for the detection of low level of virus and confirmation of the first round PCR product. PCR was carried out for 40 cycles which includes the initial denaturation and denaturation at 95°C for 3 minutes and 94°C for 1 min, respectively, followed by annealing for 1 min at 58°C, elongation for 1 min at 72°C, and final elongation at 72°C for 7 min.

Subsequently, a nested PCR was carried out by using the following: 3 *µ*l of RT-PCR product, master-mix: 14 *µ*l, forward and reverse primers: 1 *µ*l each, and RNase-free water: 6 *µ*l to make a final volume of 25 *µ*l.

The nested PCR products were examined by electrophoresis on a 1.5% agarose gel (product), stained with 10 mg/ml ethidium bromide, at 100 V for 60 mins. The 427 base pair (bp) was viewed under ultraviolet illumination. The positive PCR products were sent to DNA sequencing facility centre for sequencing, using Sanger sequencing method.

## 6. Results

A total of one hundred and sixty faecal samples from 3–5-month-old pigs collected from swine herds, one hundred and thirty from a commercial farm (UFT) located at Nkonkobe Local Municipality, under Amathole District Municipality, and thirty samples from a communal farm (UMCF) located at Mhlontlo Local Municipality, under Chris Hani District Municipality, were analysed. All the samples collected from both farms were screened for the presence of HEV: three (3) samples were positive from the samples collected from commercial farm while four (4) were positive from the samples from communal farm (Table 1).

For the seven swine HEV isolates generated from this study, nucleotide sequences of ORF 2 RT-PCR products were obtained after sequence editing, using Chromas software (version 2.5). Nucleotide sequence comparison by BLAST as implemented in the NCBI database (https://www.ncbi.nlm.nih.gov/) showed* UMCF01–04* having 87% to 99% homology with the 440 bp fragments of ORF 2 of swine HEV isolates from Japan, Netherlands, and France, respectively, while isolates* UFT01–03* showed about 82% to 93% nucleic acid identity in the 440 bp section of ORF 2 among swine and human isolates from US and Japan.

Subsequently, phylogenetic analysis of the seven isolates of swine HEV was carried out by comparing the highly preserved 300 bp of OFR 2 to other swine and human HEV strains from different geographical regions. The GenBank accession numbers of the swine and human HEV reference strains used in the phylogenetic analysis in this study are as follows: Japan: LC073306, LC022740, AB986280, AB434138, AB671034, AB671046, LC037975, AB094207, AB073911, AB094211, AB476429, AB671052, AB607892, AB094209AB112743, AB290039, AB298181, AB683185, AB607888, and AB607890; Netherlands: AF336290, AY032756, AF332620, AF336290, and AY032758; USA: AF110387, AF110388, AF466681, AF466660, and AF020497; United Kingdom: AJ344190 and AJ428851; Canada: KP255948, DQ860005, KF956531, and KP255925; Taiwan: AF117275 and KP255922; France: JQ763611; Venezuela: KJ645943; India: FJ230850; Argentina: AF264010; China: DQ445498; Philippines: HM366941; Nigeria: KJ451629 and KJ451633; South Africa: KT833800 and KU178916; Cameroun: KC012634.

**Figure 1:**
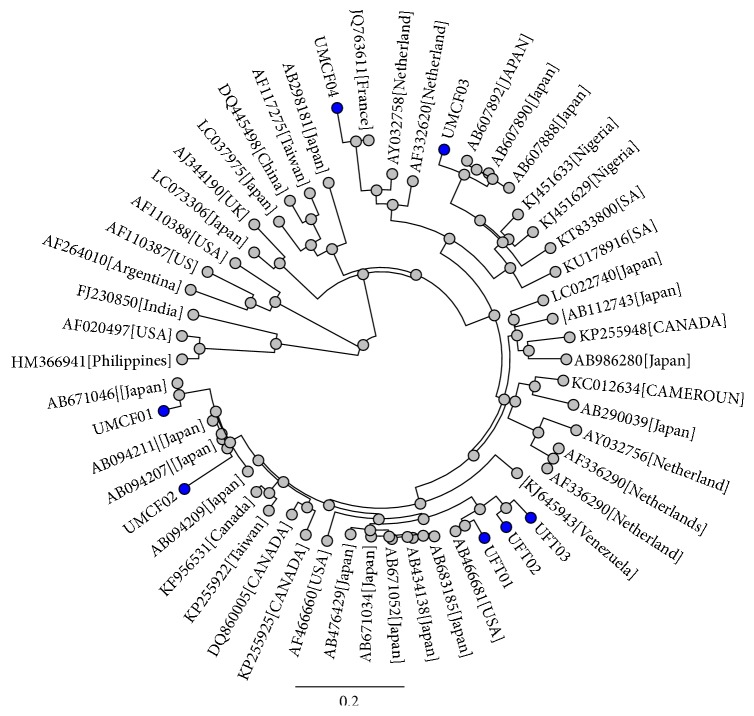
Phylogenetic relationships of HEV isolates obtained in this study. Phylogenetic analysis was based on 300 bp of the ORF 2 gene of the swine HEV isolates obtained in this study along reference sequences of other swine and human HEV strains from different geographical regions of the world that were obtained from the GenBank. Tree was drawn with Geneious R9.1.5 (Biomatters Limited). Sequences obtained in this study are given in blue dots.

Phylogenetic analysis of the swine HEV isolates was evaluated using Geneious R9.1.5 (Biomatters Limited). Phylogenetic analysis showed that all seven isolates from this study clustered with both human and swine HEV from different geographical regions of the world especially with Japan human and swine strains, Netherland swine HEV strains (AY032758, AF332620), and human HEV strain from France (JQ763611) (Figure 1). Isolates designated with* UFT01–UFT03*, recovered from commercial farm, formed a distinct cluster with swine HEV strain from United States of America (AF466681).

Isolate* UMCF01* formed a distinct cluster with the swine HEV strains from Japan (AB671046) and* UMCF02* clustered with swine HEV strains isolated from a blood donor in Japan (AB094207 and AB094211), belonging to genotype 3, while isolates UMCF03 and UMCF04 phylogenetically formed distinct clusters with both human and swine HEV strains from Japan, respectively. Sequences reported in this study have been deposited to GenBank database under the accession numbers KX896664, KX896665, KX896666, KX896667, KX896668, KX896669, and KX896670.

## 7. Discussion

Globally, large proportion of swine have been acknowledged to be infected with HEV while its recovery from the faeces of 2- to 6-month-old pigs have been reported [25, 26], and, as an emerging pathogen, several novel strains of human HEV have also been detected from patients with severe hepatitis in both developing and developed geographical regions of the world [27–29].

Various studies have reported the incidence of swine HEV from different parts of the world [30–33]. In the United Kingdom, China, Japan, and Canada, over 20% of swine faecal samples have been confirmed positive for HEV RNA [34–37], depending on the region sampled. Also, the widespread distribution of HEV has been reported in Nigeria, with prevalence rate of 76.7% of genotype 3 as well as in Cameroon, Republic of Congo, and Tunisia [8, 38]. All these are pointing to increased responsiveness and surveillance and prevalent nature of this virus in the environment. In this study, a total of 7/160 (4.4%) HEV strains, belonging to genotype 3, were recovered from swine for the first time in the Eastern Cape Province of South Africa.

In developed countries, hepatitis E virus is considered a rare infection and most hepatitis E cases are linked with travelling to endemic regions. However, in recent years, cumulative numbers of sporadic cases have been reported among adults, especially in patients with unknown epidemiological risk factor [39–41]. Also, zoonotic transmission of HEV from wild boars has recently been reported in Italy [42], as well as in Germany, where the consumption of wild deer meat was associated with indigenous HEV infection [43], and genomic sequence recovered from wild boar in Sweden showed a very high percentage of relatedness to human and domestic pigs in the same environment [44].

Besides infections in swine, the report of HEV infection in other animals is rapidly increasing. Genotypes 2, 3, and 4 strains of HEV have also been recovered in other nonhuman hosts, including mongoose, rats, birds, rabbits, bats and fish macaque, sheep, chicken, yak, and cattle [45–47]. Thus, swine has been primarily suspected as a source of zoonotic transmission owing to the fact that other animals are not closely related to human life [12, 48, 49]

Furthermore, cross-species infection has been reported in China and Korea [49], due to traditional mixed farming system whereby various domestic animals are reared together in a close range [50] and this may hypothetically proliferate the zoonotic sources which could facilitate the transmission of HEV to humans. In the study area, especially in the communal farm, it was observed that pigs are being reared with other domestic animals, including cattle, chickens, ducks, and sheep. As such, the traditional farming system portends an increased risk of zoonosis in the study area.

In South Africa, the first case of HEV genotype 3 from a 50-year-old transplant patient with a preexisting medical condition has been reported [51]. This suggests that HEV is underdiagnosed and underdocumented in this region; hence molecular HEV testing needs to be encouraged to support the management of immunocompromised patients.

The data generated from this study raises a concern about a possible zoonosis of HEV strains circulating in the study area, as farming is the occupation of majority of the populace in the study area and the use of swine waste as manure to improve the farm yields has been a common practise. Also, most of the people living in the rural parts in the study area depend on water from dams and rivers for both drinking and domestic activities, and, for this reason, transmission of HEV from animal to man is anticipated. This is consistent with the findings of [52, 53]. It is, theretofore, imperative to safeguard the public against HEV infection, especially children (<10 years), the aged people (≥50 years), immune-comprised individuals, and pregnant women. In addition, information on human HEV seroprevalence is required among the people living in the study area to establish their exposure to the virus.

The result from this study is also supported by a seroprevalence study conducted among selected group of people in relation to pork consumption in the Western Cape Province, South Africa, which revealed HEV IgG seroprevalence of 29.7% [54], suggesting swine as reservoirs of the virus.

Within respect to the sequence comparison, genetic relatedness, and phylogeny analysis of the 300 bp of ORF 2, all swine HEV strains from this study are mostly clustered with previously reported human and swine strains from USA, Netherlands, Japan, Nigeria, France, and South Africa [37, 38, 55–57].

Similar to other reports in Africa, Hepatitis E virus genotype 3 was identified and the strains characterized in this study presented a higher nucleotide homology with the strains from Japan and Netherland than the ones from Africa [38, 58–60]. The transmission of swine HEV between countries via international trade has been documented in Africa, Asia, and America [61, 62]; hence the HEV genotype 3 obtained from this study could be linked to Europe, Asia, and America, although more scientific information is required to establish this.

Swine production in South Africa, like any other countries, is practiced either on a large scale (commercial) or small scale (communal). In communal system where pigs are allowed to roam about in sourcing for food and water, they visit flowing or stagnant waterbodies, thereby contaminating it with their faeces and urine, and consequently creating a route of transmission to humans, especially in an environment where there is a close association between pigs and humans, resulting in zoonosis of HEV infection.

Subsequently, to public health concern of zoonosis, xenozoonosis is another concern among patients undergoing organ-transplant; hence as a result of adaptation in immune-compromised individuals, quasispecies, or recombination, a nonpathogenic strain of swine HEV may become pathogenic.

With the similarities of the sequences detected among the strains of the isolates from this study and the strains from Japan, Netherlands, US, and Taiwan, and from other reports from different parts of the world, it can be deduced that HEV genotype 3 is a zoonotic virus.

As it was recently reported that HEV infection is underdiagnosed, [51] had suggested that it should be included in diagnoses as it may be the cause of non-A, non-B, or non-C hepatitis case [63]. Biosafety measures should also be encouraged in surroundings near the swine herds so as to prevent the spread of swine HEV strains outside the herds, as different strains of swine HEV in circulation are observed in this study.

The present study establishes that phylogeny can be a valuable tool in determining the source of HEV infections and geographical origin of hepatitis E virus, genotype 3 strains, infecting humans. In addition, the effectiveness of this tool for tracing human infections is dependent on regular screenings of swine and other domestic animals implicated to be reservoirs of HEV. It is reasonable to assume that if more information regarding HEV strains circulating in the study was available, it would significantly increase the reliability and precision of this model for tracing infections.

Conclusively, the detection of swine HEV strains in this study linked to human HEV isolates from Japan, America, and Netherlands points toward a significant innovative track for hepatitis E virus research. From the public health perspective, priority should be given to the development of techniques for the detection of interspecies transmission at early stage. Swine HEV-mediated infection could make an animal model for hepatitis E virus studies available. It might as well prove worthwhile for vaccine development against HEV infection in humans.

In addition, molecular-based analysis of HEV occurrence in swine herds, waterbodies, and hospitals, on a regular basis, is hereby encouraged to bring about appropriate control strategies and to prevent imminent outbreak of the virus.

## Figures and Tables

**Table 1:** Age-dependent prevalence of HEV RNA in the faeces of swine in Eastern Cape Province.

Age (months)	Type of farm	Number of pigs tested	Number of pigs with HEV
3–5	Commercial	130	3 (2.3%)
3–5	Communal	30	4 (13%)

Total		160	7 (4.4%)
